# Antagonism between *Staphylococcus epidermidis* and *Propionibacterium acnes* and its genomic basis

**DOI:** 10.1186/s12864-016-2489-5

**Published:** 2016-02-29

**Authors:** Gitte J. M. Christensen, Christian F. P. Scholz, Jan Enghild, Holger Rohde, Mogens Kilian, Andrea Thürmer, Elzbieta Brzuszkiewicz, Hans B. Lomholt, Holger Brüggemann

**Affiliations:** Department of Biomedicine, Aarhus University, Aarhus, Denmark; Department of Molecular Biology and Genetics, Aarhus University, Aarhus, Denmark; Institute of Medical Microbiology, University Hospital Hamburg-Eppendorf, Hamburg, Germany; Department of Genomic and Applied Microbiology, Institute of Microbiology and Genetics, Georg-August University Göttingen, Göttingen, Germany

**Keywords:** *Staphylococcus epidermidis*, *Propionibacterium acnes*, Skin microbiota, Bacterial antagonism, Antimicrobial substance, Bacteriocin, epidermin, ESAT-6 secretion system, Polymorphic toxin

## Abstract

**Background:**

*Propionibacterium acnes* and *Staphylococcus epidermidis* live in close proximity on human skin, and both bacterial species can be isolated from normal and acne vulgaris-affected skin sites. The antagonistic interactions between the two species are poorly understood, as well as the potential significance of bacterial interferences for the skin microbiota. Here, we performed simultaneous antagonism assays to detect inhibitory activities between multiple isolates of the two species. Selected strains were sequenced to identify the genomic basis of their antimicrobial phenotypes.

**Results:**

First, we screened 77 *P. acnes* strains isolated from healthy and acne-affected skin, and representing all known phylogenetic clades (I, II, and III), for their antimicrobial activities against 12 *S. epidermidis* isolates. One particular phylogroup (I-2) exhibited a higher antimicrobial activity than other *P. acnes* phylogroups. All genomes of type I-2 strains carry an island encoding the biosynthesis of a thiopeptide with possible antimicrobial activity against *S. epidermidis*.

Second, 20 *S. epidermidis* isolates were examined for inhibitory activity against 25 *P. acnes* strains. The majority of *S. epidermidis* strains were able to inhibit *P. acnes*. Genomes of *S. epidermidis* strains with strong, medium and no inhibitory activities against *P. acnes* were sequenced. Genome comparison underlined the diversity of *S. epidermidis* and detected multiple clade- or strain-specific mobile genetic elements encoding a variety of functions important in antibiotic and stress resistance, biofilm formation and interbacterial competition, including bacteriocins such as epidermin. One isolate with an extraordinary antimicrobial activity against *P. acnes* harbors a functional ESAT-6 secretion system that might be involved in the antimicrobial activity against *P. acnes* via the secretion of polymorphic toxins.

**Conclusions:**

Taken together, our study suggests that interspecies interactions could potentially jeopardize balances in the skin microbiota. In particular, *S. epidermidis* strains possess an arsenal of different mechanisms to inhibit *P. acnes*. However, if such interactions are relevant in skin disorders such as acne vulgaris remains questionable, since no difference in the antimicrobial activity against, or the sensitivity towards *S. epidermidis* could be detected between health- and acne-associated strains of *P. acnes*.

**Electronic supplementary material:**

The online version of this article (doi:10.1186/s12864-016-2489-5) contains supplementary material, which is available to authorized users.

## Background

The microbiota of human skin is unique and complex, and is composed of a mixture of different groups of microorganisms: anaerobic, aerotolerant bacteria, such as propionibacteria; facultative anaerobic bacteria, such as staphylococci; fungi, such as *Malassezia* spp., and bacteriophages and viruses.[1, 2] According to both culture-based studies and metagenomic investigations, propionibacteria dominate in sebaceous sites and staphylococci and corynebacteria preferentially colonize moist areas [1–3]. However, different genera and species live in close proximity, in particular the two predominant skin species *Staphylococcus epidermidis* and *Propionibacterium acnes. S. epidermidis* colonizes various areas of the skin and is considered to be a skin commensal, but it can act as an opportunistic pathogen when it breaches the skin surface and enters the bloodstream [4, 5]. *P. acnes* resides mainly in the pilosebaceous skin follicles; despite being a commensal with potential health-beneficial effects [6], evidence exists that the bacterium can also be an opportunistic pathogen, e.g., in postoperative infections [7, 8]. The interaction between *S. epidermidis* and *P. acnes* and its relevance for skin health and disease of the host is largely unknown. This microbial interplay, for instance mediated through molecules involved in intercellular competition or communication, may have an impact on the fine balance of the skin ecosystem. A disturbed balance (dysbiosis) can impact skin health and might initiate or support the events that lead to skin disorders [9]. One of such disorders is acne vulgaris, a multifactorial disease of pilosebaceous units of the skin that affects adolescents [10]. Microorganisms have been associated with this disease, in particular *P. acnes*, and it has been reported that *S. epidermidis* and *P. acnes* can be isolated together from acne-affected sebaceous follicles [11].

Both species, *P. acnes* and *S. epidermidis*, are multiphyletic; *P. acnes* exhibits very limited strain- and cluster-level variation, whereas *S. epidermidis* strains and clades are more heterogeneous [12, 13]. Phylogenetic analyses can separate the *P. acnes* population into the clades I, II and III, which can be further subdivided into subclades and clonal complexes [12, 14–16]. Recently, the potential importance of *P. acnes* strain variation has been highlighted: certain lineages of *P. acnes*, in particular those of the I-1a cluster, are associated with acne, while other lineages are associated with healthy skin or deep tissue infections [12, 16, 17].

Here, we investigated the antagonistic interactions between *P. acnes* and *S. epidermidis*, including strains of both species that have been isolated from healthy and acne-affected skin. Our study revealed various levels of antagonism between *S. epidermidis* and *P. acnes* and highlighted the importance of strain-level variation. Genome sequencing of inhibitory *S. epidermidis* strains revealed an arsenal of fitness and competition functions encoded in the accessory genome. One potent strain possessed the epidermin biosynthesis cluster, and another strain harbours a functional type VII secretion system that might confer antimicrobial activity.

## Results

### Selection of bacterial strains and principal methodology

Bacterial isolates, 77 and 24 strains for *P. acnes* and *S. epidermidis*, respectively, were previously obtained by swab sampling from human skin of acne patients and healthy individuals [12]. Some additional strains were obtained from the CCUG strain collection (Additional file 1 and Additional file 2). All *P. acnes* strains were phylogenetically analysed by multi-locus-sequence typing (MLST), using the scheme of Lomholt and Kilian [12]. The *P. acnes* isolates covered all major clades (I, II, III) and all sequence types assigned by Lomholt and Kilian [12].

Simultaneous antagonism assays were carried out. In the first set of experiments, the antimicrobial activity of *P. acnes* against *S. epidermidis* was investigated: strains of *S. epidermidis* were suspended on the agar surface, thus representing the indicator strains, and *P. acnes* was added as stab culture. In the second set of experiments, in order to assess the antimicrobial activity of *S. epidermidis*, *P. acnes* isolates were used as indicator strains in a similar set-up.

### *P. acnes* strains from the phylogroup I-2 displayed increased antimicrobial activity against *S. epidermidis*

A total of 77 *P. acnes* strains were screened for their antimicrobial activity against 12 *S. epidermidis* strains. A positive inhibitory effect was defined as a zone of inhibition around the *P. acnes* stab culture of at least 2 mm. The *P. acnes* strains displayed varying degrees of antimicrobial activity against *S. epidermidis*. Among the *P. acnes* strains are potent inhibitors of *S. epidermidis*, such as strains 2.3.A1 (I-2, ST35) and 27.1.A1 (I-2, ST38) that inhibited 9 out of 12 *S. epidermidis* strains. Other strains had little inhibitory effect, such as strains 5.1.R1 (II, ST52), CCUG33951 (II, 48) and CCUG50480 (I-1a, ST6) that inhibited only 1 out of 12 *S. epidermidis* strains (Additional file 3).

One phylogroup, I-2, represented by 14 strains, displayed a significantly higher frequency of antimicrobial activity against *S. epidermidis* than the other phylogroups (I-1a, I-1b, III and II). *P. acnes* I-2 strains inhibited on average 61 % of the tested *S. epidermidis* strains, whereas strains of the other *P. acnes* types inhibited on average only 29 % of the tested *S. epidermidis* strains (Table 1). The reason for the broader antimicrobial activity of I-2 strains, i.e., inhibiting a higher proportion of the tested *S. epidermidis* strains, is likely to be found in the genomic regions that are specific for I-2 strains. On the accessory genome of type I-2 strains a genomic island is located that encodes a thiopeptide similar or identical to berninamycin A of *Streptomyces bernensis* [18, 19].

**Table 1:** Antimicrobial activity of different phylotypes of *P. acnes* against 12 *S. epidermidis* strains

Phylogroup	Antimicrobial activity in %^a^
I-1a (43 strains)	32 %
I-1b (4 strains)	42 %
I-2 (14 strains)	61 %
III (2 strains)	21 %
II (14 strains)	20 %

^a^explanatory example: *P. acnes* I-1a strains inhibited in average 32 % of the tested *S. epidermidis* strains, whereas I-2 strains inhibited in average 61 % of the tested *S. epidermidis* strains

No difference in prevalence and intensity was noted in the antimicrobial activity of *P. acnes* strains isolated from normal and acne-affected skin (Table 2). Among the I-1a strains, isolates from acne patients were not distinct in their anti-*S. epidermidis* activity from isolates obtained from healthy skin. This suggests that a particular antimicrobial activity of *P. acnes* against *S. epidermidis* does not play a role in the acne-associated microbiota.

**Table 2:** Origin of *P. acnes* strains and their antimicrobial activity against *S. epidermidis*

Skin status	Antimicrobial activity in %
healthy skin (22 strains)	38 %
light acne (17 strains)	34 %
moderate acne (11 strains)	39 %
severe acne (6 strains)	29 %

### Strain-dependent antimicrobial activity of *S. epidermidis* against *P. acnes*

**Fig. 1:**
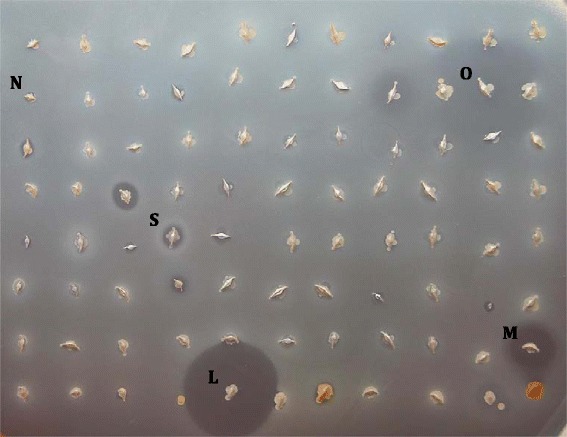
Simultaneous antagonism assay of *S. epidermidis* against *P. acnes*. A representative result of a simultaneous antagonism assay is shown. A large TS agar plate was floated with a suspension of a *P. acnes* indicator strain, and strains of *S. epidermidis* were point-inoculated on the agar surface. Growth inhibition around several of the *S. epidermidis* colonies is apparent. Diameters of the depicted inhibitory zones ranged from 3 mm to 20 mm. Arrows point to the individual zones of inhibition, i.e., opaque (O), small (S), medium (M), large (L) zones, or no inhibition zone (N)

The results of the antagonism assay were then analysed from the perspective of the antimicrobial activity of *S. epidermidis*. Eleven out of 20 *S. epidermidis* strains displayed varying degrees of antimicrobial activity against 25 *P. acnes* strains (Table 3). Among the *S. epidermidis* strains with an elevated antimicrobial activity, differences in inhibition zone diameter and appearance were observed, indicating that the antimicrobial substances were of different nature (Fig. 1). Most of the *S. epidermidis* strains displayed small zones of inhibition (2- 4 mm) against *P. acnes* and some strains produced opaque zones of inhibition. Interestingly, one strain, FS1, produced very large inhibitory zones (>10 mm), but was not active against all *P. acnes* strains tested. Another strain, 14.1.R1, was able to inhibit all *P. acnes* strains, but produced small zones of inhibition (2-5 mm). No difference in prevalence and intensity was noted in the antimicrobial activity of *S. epidermidis* strains isolated from normal and acne-affected skin, respectively (Table 3). Likewise, the origin of *P. acnes* strains did not determine their susceptibility to the antimicrobial activity of *S. epidermidis*, since strains isolated from acne lesions and healthy skin, respectively, were not significantly different in their susceptibility patterns towards *S. epidermidis* (Additional file 4).

**Table 3:** Antimicrobial activity of 20 *S. epidermidis* strains against 25 *P. acnes* strains

*S. epidermidis* strain	Isolated from	Antimicrobial activity in %^a^
**14.1.R1**	upper back, light acne	100
**AU40**	healthy alar crease	88
**AU21**	healthy nares	72
**FS1**	face, light acne	68
**GS3**	face, moderate acne	64
**AU23**	healthy nares	64
**AU48**	healthy nares	28
**AU39**	healthy alar crease	12
**AU60**	healthy nares	8
**AS1**	alar crease, light acne	8
**AU36**	healthy nares	4
**AU10, AU16, AU24, AU35, AU44, AU53, AU73, AU81, IS2**	diverse, see Additional file 2	0

^a^Listed from most potent to least potent strain, e.g., 100 % refers to an antimicrobial activity of *S. epidermidis* against all 25 *P. acnes* strains

### Antimicrobial activities of *S. epidermidis* strains 14.1.R1 and FS1

To further characterize the observed broad antimicrobial activity of *S. epidermidis* strain 14.1.R1, the screening was extended with 29 additional *P. acnes* strains representing all phylogroups. All additional *P. acnes* strains were also inhibited by this *S. epidermidis* strain (Additional file 5). To test whether the antimicrobial activity of *S. epidermidis* strain 14.1.R1 was *P. acnes*-specific, other Gram-positive and Gram-negative bacteria were also screened. *S. epidermidis* strain 14.1.R1 did not have the ability to inhibit any of the other bacterial species tested, except for a *Propionibacterium granulosum* strain. This shows that the antimicrobial activity of strain 14.1.R1 is *P. acnes*-specific, and might be active against closely related propionibacteria as well, but not against species of other genera.

Strain FS1 produced the largest inhibition zones of all tested *S. epidermidis* strains. It was particularly active against *P. acnes* type II and III strains (inhibition zone >10 mm). A diffuse, smaller inhibition zone against most *P. acnes* type I strains was observed (data not shown).

### Comparative genomics of *S. epidermidis* highlights core genome diversity

**Fig. 2:**
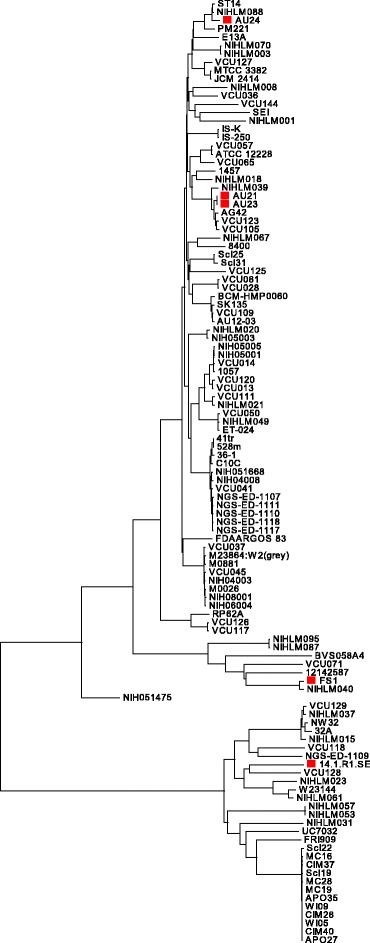
Phylogenetic tree of 112 *S. epidermidis* strains based on the shared core genome. Phylogenetic analysis the core genome sequence of approximately 1.7 Mb divides the *S. epidermidis* strain population in two distinct clades. The sequenced strains used in the antagonism assay are highlighted in red

In order to investigate the genetic basis for the antimicrobial activity of *S. epidermidis* against *P. acnes*, strains with broad (14.1.R1), medium (FS1, AU21, AU23) and no (AU24) inhibitory activities were selected and genome sequenced. One strain (AU23) was sequenced with high quality by PacBio technology and the three other isolates (FS1, AU21, AU24) were Illumina-sequenced. The genome sequence of the strain 14.1.R1 was taken from GenBank (accession number AGUC00000000; 2,555,725 bp in 131 contigs). The genome of strain FS1 has a similar size with 2,551,451 bp (in 91 contigs). The genomes of strains AU21 (2,421,814 bp in 72 contigs) and AU24 (2,429,243 bp in 61 contigs) are considerably smaller compared to 14.1.R1 and FS1, on average 130 kb. The genome size of strain AU23 is 2,487,973 bp (in 5 contigs); one contig of 42 kb represents a plasmid. To assess the phylogenetic distance of the five isolates and to relate them to other *S. epidermidis* strains, a phylogenetic tree was constructed based on the core genome of 112 *S. epidermidis* genome sequences (Fig. 2). The size of the core genome is approximately 1.7 Mb, which is in concordance with previous findings [13]. The tree shows that the *S. epidermidis* population is divided into two main clades, with a large heterogeneity within each clade. The core genomes of AU21 and AU23 are almost identical, and they group together in the same clade as strain AU24. Strain FS1 belongs to the same clade, but is located in an outlier cluster, indicating increased phylogenetic distance to strains AU21, AU23 and AU24. The strain 14.1.R1 is phylogenetically most distinct and clusters in the second clade. Thus, antimicrobially active *S. epidermidis* strains are scattered in phylogenetically not closely related clades. This indicates that the inhibitory activity of *S. epidermidis* against *P. acnes* is not part of the core genome, but is rather encoded in the accessory genome.

### The accessory genome encodes diverse fitness functions including factors involved in interbacterial competition

In order to identify the genomic basis for the antimicrobial activity against *P. acnes*, we focussed on genomic regions in strains AU21, AU23, FS1 and 14.1.R1 that are absent in strain AU24, the isolate without inhibitory activity. Comparative genome analysis revealed that all strains comprise several mobile genetic elements that are only rarely found in other sequenced *S. epidermidis* strains, and that confer a variety of fitness functions, including antibiotic and stress resistance, biofilm formation, interbacterial competition and, possibly, virulence. A comprehensive analysis was done for each strain:

#### AU21 and AU23

Strains AU21 and AU23 had similar moderate antimicrobial activities against *P. acnes,* and produced diffuse inhibitory zones. The accessory genomes of both strains are largely identical, underlining their close phylogenetic relationship. The accessory genome of strains AU21 and AU23 is distributed in six larger regions, that are absent in the chromosome of strain AU24 (Additional file 6A and Additional file 7A). A variety of functions can be found. One region (region 1 in Additional file 6A) is rarely found in any other sequenced *S. epidermidis* genome; it is a genomic resistance island of possible phage origin, encoding FusB that confers resistance to the antibiotic fusidic acid. This island has recently been described in *S. epidermidis* strain NTUH-3692 [20]. Another genomic island (region 3) encodes proteins conferring increased tolerance to divalent metal ions such as zinc, copper and cobalt. Region 5 encodes an arginine deiminase (ADI) system, catalyzing the conversion of arginine to ornithine via citrulline, thereby generating NH_3_ and ATP. The ADI system is used by several bacterial species to facilitate survival in acidic conditions [21]. At least two other islands (region 2 and 4) encode proteins related to the bacterial surface, such as teichoic acid modifying enzymes and the cell surface proteins SasG and SasC that are implicated in host interactions and biofilm accumulation [22]. The 42 kb plasmid (region 7) encodes among others several transport systems, a putative lysine (or arginine) biosynthesis gene cluster, and proteins protective against oxidative stress such as glutathione peroxidase/reductase (Additional file 7A).

The shared accessory genome of AU21 and AU23 encodes no known bacteriocin or any other previously identified antimicrobial system. We conclude that the inhibitory activity of these strains is conferred either by an unknown bacteriocin or bacteriocin-like substance, or by other means, such as the secretion of the fermentation product succinic acid, which has been shown to inhibit *P. acnes* [23]. In this regard, the ADI system found in AU21 and AU23 might increase self-protection of succinic acid-producing cells from the adverse effects of acidification.

#### FS1

Strain FS1 produced the largest zones of inhibition against most *P. acnes* strains. Its genome harbors 10 genomic regions that are absent from strain AU24 and most other sequenced *S. epidermidis* strains, including the three examined strains AU21, AU23 and 14.1.R1 (Additional file 6B and Additional file 7B). Several regions encode phage-related proteins (regions 3, 4, 8 and partially 10 in Additional file 6B). Region 8 has similarity to *S. aureus* superantigen-encoding pathogenicity islands (SaPIs). These phage-related chromosomal islands are considered to be phage satellites or parasites [24]. Another island (region 6) represents the well-characterized biofilm operon *icaADBC* that encodes the polysaccharide intercellular adhesin (PIA) [25]. Two genomic regions are interesting with regards to antimicrobial activity. Region 1 represents a full or partial plasmid, as judged from the presence of genes encoding plasmid replication proteins and a toxin-antitoxin system conferring plasmid stabilisation (Additional file 7B). This 42 kb region harbors an operon encoding a bacteriocin, belonging to the lactococcin-972 superfamily (Pfam09683), an immunity protein as well as a putative bacteriocin export ABC transporter. The other genomic region encoding an antimicrobial substance is a 12 kb cluster located in region 10. It encodes EpiA, the precursor peptide for the well-characterized lantibiotic epidermin, as well as proteins involved in posttranslational modification of EpiA, including homologs of EpiBCDRP [26]. Epidermin has been shown to act on several Gram-positive bacteria, including *P. acnes*. Interestingly, *epiP* is frameshifted in the genome of FS1, resulting in a truncated protein. EpiP is the precursor-processing protease that cleaves the 52-aa precursor peptide into the mature 22-aa bioactive peptide [27]. This raises doubts about the activity of the epidermin(-like) peptide in strain FS1. Biosynthesis of epidermin is encoded on a 54 kb plasmid in *S. epidermidis* strain Tü3298 [26]. Thus, it is likely that the 12 kb *epiA* containing contig (region 10) and the 42 kb plasmid contig (region 1) together constitute a 54 kb plasmid. In summary, the antimicrobial activity of strain FS1 likely derives from one or both identified bacteriocins.

#### 14.1.R1

**Fig. 3:**
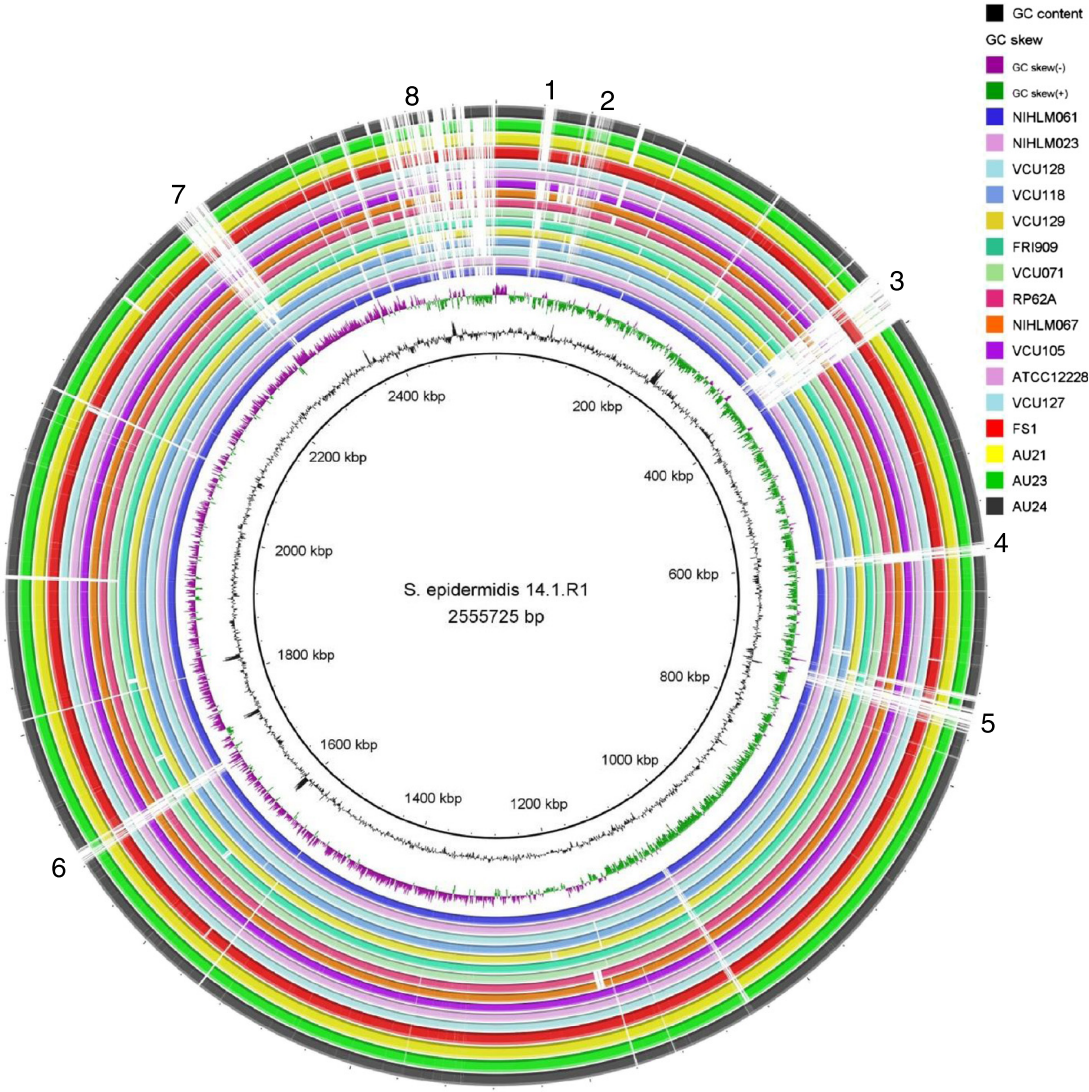
Genome comparison of 17 *S. epidermidis* strains. Strain 14.1.R1 was used as the reference genome. The four newly sequenced genomes of strains FS1, AU23, AU21 and AU24 are represented by the four outermost rings. The other 13 genomes were selected based on their position in the phylogenetic core genome tree, which shows the clustering of strains VCU128, NIHLM023, NIHLM061, VCU118 and VCU129 with strain 14.1.R1 (Fig. 2). Regions of genomic variability (1-8) can be detected (see Additional file 7C for their gene content). The BRIG program was used to generate the figure

Next, we analysed the genome of *S. epidermidis* 14.1.R1, the strain with extraordinary antimicrobial activity against *P. acnes*. The genome comparison revealed eight large genomic islands, some of which are only found in strain 14.1.R1 (Fig. 3). Signatures of their horizontal acquisition can be detected in these clusters, such as an aberrant GC content and/or GC skew, and mobility-associated functions including replication proteins and integrases (Fig. 3 and Additional file 7C). Region 1 encodes antibiotic resistance determinants (beta-lactamase) and a recombinase system similar to the Staphylococcal Cassette Chromosome *mec* (SCC*mec*). Region 2 encodes an alternative secretion system, the accessory Sec system (SecA2-SecY2) that is dedicated to the export of a family of glycosylated serine-rich repeat proteins [28]. Region 3 constitutes a prophage genome that is also present in *Staphylococcus capitis*. Regions 4 and 6 represent phage-related chromosomal islands with similarity to SaPIs [24]. The SaPI-like region 4 harbours the putative virulence gene *vapE* as previously noted in the literature [20]. Region 5 harbors a large gene (4.6 kb) encoding a biofilm-associated protein (Bap) that has been shown to be important in biofilm formation independent of the PIA/PNAG exopolysaccharide [29]. Region 7 is described below in more detail. Region 8 is composed of 7 different contigs varying between 3 and 14 kb in size. Some of these contigs appear to be plasmids. Most *S. epidermidis* strains contain plasmids; one to six plasmids are present in the strains that have been completely sequenced to date. Strain 14.1.R1 contains most likely several plasmids. For example, a 14 kb contig (AGUC01000118) has similarity to *S. epidermidis* plasmid SAP110A (NC_013383) that encodes antibiotic resistance functions.

### *S. epidermidis* strain 14.1.R1 harbors the ESAT-6 secretion system

**Fig. 4:**
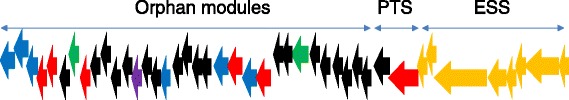
Genomic locus in *S. epidermidis* 14.1.R1 encoding an ESAT-6 secretion system and multiple nuclease/immunity protein pairs. In orange, ESAT-6 secretion system (ESS) and secreted effectors (from right to left: *esxA, esaA, essA, esaB, essB, essC*, unknown effector, *esxB*), In red: proteins with DNA/RNA nuclease domains, possible toxin components; in green, SMI1/KNR4 family (SUKH-1) domain proteins, possible immunity components; lila, serine protease (NfeD-like protein); in blue, proteins with domains of unknown function (DUF600, DUF1851, DUF4467). PTS, polymorphic toxin system. See Additional file 7C for the genomic location and annotation

A 27 kb genomic region (region 7, located on contig AGUC01000069) of strain 14.1.R1 is of particular interest (Additional file 7C). This island is absent in most sequenced *S. epidermidis* strains, but a BLASTn analysis showed partial homology to *S. aureus*. The 27 kb region harbors 43 genes (Fig. 4); among them are seven genes (*esxA, esaA, essA, esaB, essB, essC,* and *esxB*) that encode components of the ‘early secretory antigenic 6 kDa’ (ESAT-6) secretion system (ESS) and ESS-secreted substrates. To our knowledge, this Sec-independent secretion system has so far not been described in any other *S. epidermidis* strain. The ESS, with similarity to the type VII secretion of *Mycobacterium tuberculosis*, has been studied in *S. aureus*, where it was found to be involved in virulence via the secretion of various factors, including EsxA, EsxB, EsaC and EsaD [30–32]. Strikingly, EsxA was detected in this study in the bioactive supernatant of *S. epidermidis* 14.1.R1 by mass spectrometry (see below), indicating that the ESS is functional and might contribute to the special antimicrobial phenotype of this strain. Besides the 12 kb ESS locus, other genes within this 27 kb region might be functionally linked to the secretion system. The genes downstream of the ESS cluster encode several polymorphic toxins with nuclease domains and associated immunity proteins. It is hypothesized that such nuclease toxins can confer contact-dependent growth inhibition (see discussion).

### Partial purification and initial characterization of the bioactive compound from *S. epidermidis* 14.1.R1

It was noted that the antimicrobial activity of strain 14.1.R1 against *P. acnes* could be obtained only when *S. epidermidis* was grown on agar plates; no significant antimicrobial activity could be detected when *S. epidermidis* was grown in liquid medium. Hence, a partial purification of the bioactive compound of strain 14.1.R1 was tried using agar plate-grown *S. epidermidis*. A concentrated cell-free supernatant (cCFS) extract was obtained from harvesting 45 agar plates. It was noted that bioactivity was rapidly lost upon dilution of the cCFS. Stability of the bioactivity was reduced when storing the cCFS at 4 °C in non-coated Eppendorf tubes, indicating non-specific adsorption of the bioactive substance to the vessel walls. Coating of the tubes with Emulphogene® could prevent the time-dependent loss of activity. Exposure of the cCFS to proteinase K resulted in a complete loss of inhibitory activity, suggesting that the active substance is of proteinaceous nature. Antimicrobial activity was unaffected following exposure to low pH (pH 2) for 2 h with subsequent neutralization. Heat treatment of the cCFS (60 °C for 10 min) led to complete loss of inhibitory activity.

### Identification of proteins in the bioactive cCFS extract of *S. epidermidis* 14.1.R1

In an attempt to identify the antimicrobial compound produced by *S. epidermidis* 14.1.R1, the cCFS containing the bioactivity against *P. acnes* was analysed by MS. The cCFS extract was separated in different concentrations by SDS-PAGE and visible bands were subject to MS analysis (Additional file 8). Two proteins, the phenol soluble modulin (PSM) β1 (MW: 4639 Da) and PSMβ2 (MW: 4644 Da) were identified in one band. Homologs of PSMβ1 and PSMβ2 were found in the genome of strain 14.1.R1 (Additional file 9). The non-cytolytic PSMβ proteins have been previously investigated; they are associated with promotion of biofilm structuring, maturation and detachment in vitro [33, 34]. It is unlikely that the non-cytolytic PSMβ proteins are responsible for the antimicrobial activity of strain 14.1.R1, since these proteins are not unique to strain 14.1.R1, but present in many *S. epidermidis* strains, including the non-inhibiting strain AU24. Moreover, the PSMβ proteins are heat-resistant [35], but we showed that the bioactive protein of strain 14.1.R1 is heat-labile.

A second band of the SDS-PAGE gel was identified as EsxA (HMPREF9956_2246) (Additional file 10). This protein is encoded in the ESS island described above, and is a known effector protein secreted by the ESS. The function of this protein in *S. epidermidis* needs to be explored in the future.

Another protein band was identified as a protein similar to DNA-binding protein HU of *S. aureus* strain Mu50 (HMPREF9956_1196) (data not shown). This histone-like protein belongs to the nucleoid-associated proteins and contributes to chromosomal compaction and maintenance of negative supercoiling.

Taken together, these data indicate that *S. epidermidis* strain 14.1.R1 secretes putative virulence factors such as PSMs and EsxA. The fact that EsxA was detected indicates that the ESAT-6 secretion system is functional in *S. epidermidis* 14.1.R1.

## Discussion

Interspecies competition is a common phenomenon among members of the (human) microbiota; it can be conferred by several means. Prevalent are secreted antimicrobial substances of the bacteriocin family that can kill competing bacteria with different mechanisms, for example by pore-forming, nuclease, or cell wall synthesis-interfering activities. In addition, primary or secondary metabolites also impact on the microenvironment of the producing cell; this can result in the inhibition of competing bacteria in the close vicinity.

This study examined the antimicrobial action of *P. acnes* against *S. epidermidis* and vice versa as determined by an in vitro simultaneous antagonism assay. In agreement with previous findings, bacterial antagonism between *P. acnes* and *S. epidermidis* is a common phenomenon [23, 36]. Our study revealed a higher frequency of antimicrobial activity among *P. acnes* strains belonging to the I-2 phylogroup as compared with other phylogroups of *P. acnes* against *S. epidermidis*. A likely possibility is the presence and secretion of a bacteriocin or bacteriocin-like substance specific to phylogroup I-2. A genomic comparison previously identified some phylogroup I-2-specific genes [18, 19]. Among these, a thiopeptide biosynthesis gene cluster located in a genomic island is present in all genomes of I-2 strains. Transcriptome analyses of *P. acnes* have highlighted the activity of this genomic island in the I-2 phylogroup of *P. acnes* [18]. Two thiopeptide precursor peptides are encoded in the *P. acnes* thiopeptide gene cluster; one precursor possesses the 16-aa structural peptide of berninamycin A from *Streptomyces bernensis* [37]. We therefore assume that the *P. acnes* thiopeptide has structural similarity or identity to berninamycin A. Thiopeptides such as berninamycins have been isolated from soil-derived bacteria of the *Streptomyces* genus and from marine sources, and are peptides with an antimicrobial potential that are potent inhibitors of protein synthesis in Gram-positive bacteria [38, 39]. Thus, we hypothesize that a thiopeptide produced by *P. acnes* type I-2 strains could be responsible for the more frequent antimicrobial activity against *S. epidermidis*. Future research needs to be done to confirm this hypothesis.

With regard to the antimicrobial activity of *S. epidermidis* against *P. acnes*, the current study revealed a strain-dependent pattern of antimicrobial activity among *S. epidermidis*. In agreement with the study of Holland et al. [36], we found no difference in the antimicrobial susceptibility among *P. acnes* isolates from healthy or acne-affected individuals. This might mean that susceptibility/resistance of *P. acnes* towards the antimicrobial activities of *S. epidermidis* is not important in the acne pathogenesis.

Our study showed a large variation of the antimicrobial activity among *S. epidermidis* strains against *P. acnes*. Among the inhibitory strains, the nature and size of inhibition zones varied, indicating that different antimicrobial mechanisms are at play. Comparative genome analyses revealed that there was no strong phylogenetic relatedness between inhibitory *S. epidermidis* strains. This indicates that the antimicrobial activity is not encoded on the core genome, but rather encoded on mobile genetic elements (MGEs) that are exchanged through horizontal gene transfer. Accessory traits, such as the ability to produce antimicrobial substances, have often been found to be encoded on MGEs in staphylococci [40–42].

Four strains with no, medium or broad antimicrobial activities were sequenced in order to determine the genomic basis of their respective phenotypes. Based on the sequence data different possible mechanisms conferring antimicrobial activity were identified.

1. For strains AU21 and AU23 no known bacteriocin gene was found to be encoded in the accessory genome. It is possible that these strains employ an unknown bacteriocin or an entirely novel mechanism. Alternatively, the production of metabolic products could have anti-*P. acnes* effects. A candidate is succinic acid that has been identified to be produced by *S. epidermidis* during fermentation [23]. This acid is able to kill *P. acnes* by lowering its intracellular pH. The producer strain needs to develop a self-protective mechanism against acidification. Interestingly, AU21 and AU23 carry an arginine deiminase (ADI) system that can confer such protection via the production of NH_4_. The ADI system has been shown to be crucial for resistance to acid stress in certain *S. epidermidis* strains [43]. The involvement of a metabolite such as succinic acid could also explain the diffuse nature of the inhibitory zone, which might result from incomplete lysis of *P. acnes*.
2. The strain FS1 produced large inhibition zones. Its genome contains two gene clusters encoding bactericoins; both clusters are likely to be encoded on a plasmid of 54 kb. One bacteriocin is of the lactococcin-972 superfamily and has not previously been studied to our knowledge. It is 130 aa in size and harbors an N-terminal signal peptide of 25 aa, indicative of Sec-dependent export. A putative immunity protein with transmembrane helices and a transporter are encoded immediately downstream. The second bacteriocin is a lantibiotic. The precursor peptide is identical to epidermin, a well-characterized lantibiotic of *S. epidermidis* [26]. Epidermin and the closely related gallidermin act on the cytoplasmic membrane of Gram-positive bacteria. Gallidermin was successfully tested in a topical formulation on rat skin showing antibacterial activity against *P. acnes* and *S. aureus* [44]. Due to the detected frameshift mutation of *epiP* in strain FS1, it has to be investigated in the future if the epidermin precursor peptide is not cleaved or processed differently in strain FS1.
3. Our study further revealed that the *S. epidermidis* strain 14.1.R1 had a broad inhibitory activity; it was the only tested *S. epidermidis* strain that inhibited all tested *P. acnes* strains. By a whole genome comparison approach, we searched for the underlying gene(s) responsible for the exceptional phenotype of *S. epidermidis* 14.1.R1. One genomic island was of special interest, the island was neither found in strain AU24, nor in most other genomes of sequenced *S. epidermidis* strains. Encoded on this island is a specialized ESAT-6 secretion system (ESS), which is homologous to the type VII secretion system of *M. tuberculosis* [30, 45]. This system in mycobacteria is required for full virulence as it has roles in the survival of mycobacteria in macrophages, granuloma formation, induction of apoptosis and autophagy, phagosomal rupture, and host cell lysis [30, 45–48]. The ESS has also been described in other Gram-positive bacteria, including *S. aureus* [31, 32, 45], but it has not yet been described in *S. epidermidis*. ESS-secreted effectors, belonging to the WXG100 family of proteins, whereof ESAT-6 and CFP-10 from *M. tuberculosis* are the founding members, have been identified in *S. aureus*, designated EsxA and EsxB for ESAT-6 extracellular protein A and B, respectively [31, 49–52]. No clear biological function has been attributed to the ESS effector proteins in *S. aureus* to date, but mutants that failed to secrete EsxA and EsxB displayed defects in abscess formation in mice, suggesting that these proteins are important during staphylococcal disease [31]. A recent study showed that EsxA interferes with host cell apoptotic pathways and, together with EsxB, mediates the release of intracellular *S. aureus* from the host cell [52]. The ESS is most likely functional in strain *S. epidermidis* 14.1.R1 as we could detect EsxA in the cell-free supernatant. However, it is unlikely that EsxA is responsible for the exceptional antimicrobial activity of *S. epidermidis* 14.1.R1 against *P. acnes*, since EsxA homologs have not been described as antimicrobial but rather as host cell-interacting factors involved in persistence and spread of the bacterium in the host. [31, 51, 52] The genomic island encoding the ESS has another interesting feature that could be linked to, or responsible for, the antimicrobial activity of strain 14.1.R1. Bioinformatics analysis revealed that the island contains other possible ESS effectors: The protein encoded downstream of *esxB* has an N-terminal LXG domain, a possible recognition site for a type VII secretion system [53]; its C-terminus encodes a predicted ribonuclease domain, which is a toxin component of the YeeF-YezG toxin-antitoxin module. In fact, this protein represents a polymorphic toxin; such toxins have recently been discovered to mediate interbacterial competition [53–55]. Moreover, many of the 34 genes downstream of ESS encode other toxins and immunity proteins of the SUKH superfamily. These toxin-immunity protein pairs are orphan modules, encoding alternate toxin domains and their cognate immunity proteins; such orphan modules have been found in many bacteria [53–56]. The toxin domains include DNA/RNA non-specific endonuclease (pfam13930); nuclease of the HNH/ENDO VII superfamily (pfam14414); toxin 43 superfamily (pfam15604) and serine protease (NfeD-like protein). Some polymorphic toxin systems are involved in contact-dependent growth inhibition (CDI) [56, 57]. CDI has been reported in proteobacteria; direct contact with the target cell is required to deliver nuclease toxins into target cells. Taken together, we hypothesize that a type VII-like secretion system in *S. epidermidis* 14.1.R1 is able to deliver polymorphic toxins directly onto or into target bacteria resulting in CDI. This could be a possible explanation of the broad antimicrobial phenotype of strain 14.1.R1 against *P. acnes,* with the rather small inhibitory zones produced by 14.1.R1. It would also explain why we were unable to purify the antimicrobial substance and why the anti-*P. acnes* activity was only observed when *S. epidermidis* 14.1.R1 was grown on agar plates.

Further studies are required to assess the in vivo relevance of the anti-*P. acnes* activity exerted by many *S. epidermidis* strains. The method used in this study is an in vitro antagonism assay. The detectable inhibitory activity is dependent on growth conditions such as the medium composition and the applied incubation conditions (e.g., oxygen levels). Therefore, it is not clear to what degree the inhibition phenomena obtained on agar medium reflect the in vivo settings on human skin. It further needs to be clarified if the antimicrobial activity results in a competitive advantage of the producing strain in vivo and how this impacts on the skin ecosystem.

## Conclusions

This study examined antagonistic interactions of two prominent members of the skin microbiota, *P. acnes* and *S. epidermidis*. The study showed a variety of clade- and even strain-specific inhibitory activities. Genome sequencing of selected *S. epidermidis* isolates highlighted the heterogeneity of this species, and the presence of multiple genomic islands. Accessory genome analyses identified several possible inhibitory mechanisms, including the secretion of acidic fermentation products and the respective self-protection via ADI, the production of bacteriocins such as epidermin, and the employment of a type VII-like secretion system (ESS) that might deliver polymorphic toxins on or into the target cell. The functionality of this system in *S. epidermidis* is likely, as judged from the identification of an ESS effector in the culture supernatant.

As a possible application, it could be envisaged that the diverse anti-*P. acnes* activity of *S. epidermidis* could be exploited, for example as a probiotic treatment approach against acne vulgaris or other *P. acnes*-associated diseases.

## Methods

### Bacterial strains

*S. epidermidis* and *P. acnes* strains originating from healthy and acne-affected individuals were obtained by skin swabs using cotton sticks. The strains were previously collected by Lomholt and Killian [12]. All strains were previously identified by 16S rRNA amplicon sequencing, and, for the *P. acnes* isolates, further typed by multi-locus sequence typing (MLST) [12]. Detailed information about the origin and the phylotypes of the strains used in this study is given in Additional file 1 and Additional file 2.

Bacteria were cultivated on 5 % sheep blood agar medium for one (*S. epidermidis*) or three (*P. acnes*) days. *S. epidermidis* was cultivated at 37 °C aerobically, supplemented with 5 % CO_2_. *P. acnes* was cultivated at 37 °C in an anaerobic chamber, in an atmosphere of 80 % N_2_, 10 % CO_2_ and 10 % H_2_.

### Agar plate antagonism assay to screen for antimicrobial activity

*P. acnes* strains were individually screened for antimicrobial activity against *S. epidermidis* indicator strains. The screening procedure was carried out as a simultaneous antagonism assay, as described by Tagg and Bannister, with slight modifications [58]. In brief, the screenings were carried out on large Trypticase Soy (TS) agar plates (14 cm diameter) by the following procedure: 150 μl liquid TS broth cultures of individual *P. acnes* strains was aliquoted into wells of a flat-bottom 96-well microtiter plate. TS agar plates were floated with a lawn of the individual *S. epidermidis* (suspended in 0.85 % NaCl) indicator strains. The concentrations of the indicator strain suspensions were standardized by comparison to a 0.5 McFarland standard (150 × 10^6^ CFU/ml, OD = 0.125). The plates were allowed to dry in a laminar flow bench for 15 min. The *P. acnes* strains were then simultaneously point-inoculated with an in-house developed multi-inoculator device onto the surface of the TS agar plates. The plates were incubated at 37 °C for 72 h, in an anaerobic chamber, in an atmosphere of 80 % N_2_, 10 % CO_2_ and 10 % H_2_, and examined for zones of growth inhibition.

The same approach was used to screen *S. epidermidis* strains for their antimicrobial activity against *P. acnes* indicator strains. TS agar plates were floated with a lawn of the individual *P. acnes* indicator strains. 20 *S. epidermidis* strains were simultaneously point-inoculated onto the surface of the TS agar plates and the plates were incubated at 37 °C for 72 h in an anaerobic chamber. In addition, plates were incubated in microaerophilic conditions, using the GasPak system (Oxoid, AnaeroJar) with CampyGen sachets. Each antagonism assay was carried out in triplicates.

### Building a *S. epidermidis* core genome phylogenetic tree

Genome sequences of 112 *S. epidermidis* strains were used to build a phylogenetic tree based on all shared sequences (i.e., the core genome of the species). Genomes were downloaded from NCBI Genome and WGS databases, with the exception of strains AU21, AU23, AU24 and FS1 that were sequenced for this study. The core genome was identified by slicing the genome sequence of strain 14.1.R1 into fragments of 200 bp which were used as query sequences in BLASTn (BLAST+) [59] to extract homologous sequences from the other 110 genomes. Default parameters of BLASTn were used together with a 65 % cut-off on coverage. Homologous sequences of each fragment were aligned using Muscle [60] and subsequently all fragments were concatenated into one sequence per strain using a python script. The core genome phylogenetic tree was built in Mega v6 [61] using the minimal-evolution algorithm with pair-wise deletion and 100 bootstrap replications. A total of 139,881 single nucleotide polymorphisms were found.

### Genome sequencing

Genomic DNA from *S. epidermidis* strains AU21, AU23, AU24 and FS1 was isolated using the MasterPure Gram-positive DNA Purification Kit (EpiCentre MGP04100) according to the manufacturer’s instructions. The purity and quality of the gDNA were assessed on a 1 % agarose gel and with a nanodrop apparatus. For strains AU21, AU24 and FS1, library preparation and Illumina sequencing were carried out at the Göttingen Genomics Laboratory, Germany. Sequences were trimmed with Trimmomatic 0.32 (http://www.usadellab.org/cms/?page=trimmomatic) and assembled with SPAdes 3.1.1 (http://bioinf.spbau.ru/en/spades), resulting in 2,421,814 bp in 72 contigs (AU21), 2,429,243 bp in 61 contigs (AU24), and 2,551,451 bp in 91 contigs (FS1), respectively. The GenBank accession numbers of the draft genome sequences are LNUN00000000 (AU21), LNSW00000000 (AU24) and LOAT00000000 (FS1).

The genome of *S. epidermidis* AU23 was sequenced on a PacBio RS I sequencing instrument using an 10 kb insert library (GATC Biotech AG, Germany), resulting in a total of 66,762 post-filtered continuous long reads with an average length of 3,687 bp. A sequence coverage of approximately 81-fold was obtained after sequence read assembly, that resulted in a genome size of 2,487,973 bp split into 5 contigs. The GenBank accession number of the draft genome sequence of strain AU23 is LNUS00000000.

### Gene prediction, annotation and comparative genome analysis

For *S. epidermidis* strains AU21, AU23, AU24, and FS1 open reading frames (ORFs) and tRNAs were identified and annotated using the RAST server [62]. Additional manual annotation of ORFs of interest was performed on the basis of homology searches with the public non-redundant (nr) database using BLASTp. Domain search was done using PFAM (http://pfam.xfam.org/). For comparative genome analysis, the program BRIG was used [63]. The following genomes of *S. epidermidis* were taken from GenBank to build a BRIG genome comparison: 14.1.R1 [BioProject: PRJNA64683], VCU128 [BioProject: PRJNA53771], VCU118 [BioProject: PRJNA53755], VCU129 [BioProject: PRJNA53773], VCU071 [BioProject: PRJNA53733], VCU105 [BioProject: PRJNA53739], VCU127 [BioProject: PRJNA53769], RP62A [BioProject: PRJNA64], NIHLM023 [BioProject: PRJNA62371], NIHLM061 [BioProject: PRJNA62355], NIHLM067 [BioProject: PRJNA62353], FRI909 [BioProject: PRJNA48603], ATCC12228 [BioProject: PRJNA279].

### Partial purification of bioactive fraction from *S. epidermidis* 14.1.R1

*S. epidermidis* strain 14.1.R1 was cultivated on 45 TS agar plates containing 0.1 % (w/v) skim milk at 37 °C in an aerobic atmosphere supplemented with 5 % CO_2_ for 24 h. Bacteria were harvested from all plates and suspended in 225 ml 0.9 % NaCl solution. The bacteria were then removed by centrifugation at 5000 g for 30 min at room temperature, followed by sterilization of the supernatant using 0.22 μm filter disks (Sarstedt). The cell-free supernatant (CFS) was concentrated 75-fold by centrifugal ultrafiltration on a Centriprep® centrifugal filter unit (Millipore) at 3000 g at 4 °C. The protein content in the concentrated CFS (cCFS) was measured by UV absorption at 280 nm. To assay for the potency of cCFS, multiple dilutions were made and tested for bioactivity against a sensitive *P. acnes* indicator strain by the spot-on-lawn assay; a volume of 10 μl cCFS was spotted onto the centre of a pre-dried 5 % sheep blood agar plate seeded with a suspension of a *P. acnes* indicator strain (0.5 McFarland standard: 150 × 10^6^ CFU/ml, OD_550nm_ = 0.125). The plates were incubated at 37 °C in an anaerobic chamber, in an atmosphere of 80 % N_2_, 10 % CO_2_ and 10 % H_2_, for 48 h. A clear zone of growth inhibition surrounding the spotted samples indicated antimicrobial activity.

### Effect of proteinase K, pH, heat treatment and storage on antimicrobial activity

The sensitivity of the cCFS to proteolytic activity was tested, employing a 2 h treatment with proteinase K at 37 °C at a final concentration of 10 mg/ml. After incubation, the cCFS was assayed for residual activity. In order to determine the effect on pH on bioactivity, the pH of the cCFS was adjusted to pH 2 using 1 M HCl and incubated at RT for 2 h, before neutralizing. The cCFS was subsequently assayed for residual activity. To determine the thermal stability, the cCFS was heated at 60 and 80 °C for 10, 30 or 60 min, respectively, after which each sample was assayed for residual activity. The cCFS was stored at -20 °C and 4 °C. At different time intervals, between 24 h and 120 days, the cCFS was assayed for residual activity. Containers used in the following experiments were coated with Emulphogene® (Polyoxyethylene 10 Tridecyl Ether, Sigma-Aldrich) in order to prevent binding of the active substance to the container surface.

### Gel electrophoresis and mass spectrometry

The cCFS was subjected to protein identification. Since most antimicrobial substances are low molecular weight (LMW) compounds we focussed on the analysis of LMW proteins, using tricine-sodium dodecyl sulphate-polyacrylamide gel electrophoresis (tricine-SDS-PAGE) [64]. Polyacrylamide concentration in the separating gel was 18 %. Electrophoresis was conducted at a constant voltage of 50 V for 30 min, followed by 120 V for 4 h. A polypeptide SDS-PAGE molecular weight standard (Bio-Rad) with sizes ranging from 1.06 to 26.6 kDa was included. After electrophoresis the gel was silver stained and bands were selected for Matrix-Assisted Laser Desorption/Ionization Quadrupole Time-Of-Flight (MALDI-Q-TOF) Mass Spectrometry (MS). Individual protein bands were excised from the gel, destained and subjected to in-gel tryptic digestion as described previously [65]. The extracted peptides were desalted using C_18_ Stage Tips (Proxeon, Thermo Scientific) and the adsorbed peptides were eluted directly onto a Bruker Autoflex MSP 96 Stainless Steel Target Plate using 1 μl MALDI-matrix solution (2 mg/mL α-cyano-4-hydroxy-cinnamic acid (CHCA), 750 μl acetonitrile, 248 μl dH_2_O, 2 μl trifluoroacetic acid). The solvent was allowed to evaporate leaving matrix crystals in which the tryptic peptides were embedded. The mass-to-charge (*m/z*) ratios of eluted peptides were analysed on a MALDI-Q-TOF Mass Spectrometer (Micromass Q-Tof Ultima Global, Walters). Fibrinopeptide B (MW: 1570.6 Da) was used as an internal standard for all obtained MS data. All generated MS and MS/MS data were processed using the Mascot software [66] (www.matrixscience.com). For peptide mass fingerprinting (PMF), mass spectra were acquired in the positive-ion mode over the range 700-3500 *m/z*. The search parameters allowed for variable modifications of methionine oxidation and fixed modifications of cysteine propionamide adduct. There was a peptide mass tolerance of 1.2 Da, and a single missed tryptic cleavage was allowed in the search. The generated MS/MS data were examined using the MS/MS Ion Search option in Mascot, with a fragment mass tolerance of 0.6 Da, and allowance of 1 missed cleavage. Proteins were identified via database queries with the search engine Mascot in three databases (NCBI-nr, Swiss-Prot and Mass Spectrometry Protein Sequence Database (MSDB)). The MS data were also searched directly against the proteome of *S. epidermidis* 14.1.R1. The probability score calculated by the software was used as the criterion for correct identification [66].

## Ethics approval and consent to participate

The study protocol was approved by the Ethics Committee of the County of Aarhus, and the study was conducted according to the principles of the declaration of Helsinki. Written informed consent was obtained from study participants and/or their legal guardians.

## Availability of data and materials

The Whole Genome Shotgun projects of *S. epidermidis* strains have been deposited at DDBJ/EMBL/GenBank under the accession numbers: LNUN00000000 (AU21), LNUS00000000 (AU23), LNSW00000000 (AU24) and LOAT00000000 (FS1). Other supporting data are included as Additional files.

## Additional files

Additional file 1:
***P. acnes*** **strains used in this study.** (DOCX 35 kb) Additional file 2:
***S. epidermidis*** **strains used in this study.** (DOCX 17 kb) Additional file 3:
**Inhibitory activity of 77** ***P. acnes*** **strains against 12** ***S. epidermidis*** **strains.** (DOCX 31 kb) Additional file 4:
**Sensitivity of individual** ***P. acnes*** **strains against the antimicrobial activity of 20** ***S. epidermidis*** **strains.** (DOCX 16 kb) Additional file 5:
**Antimicrobial activity spectrum of** ***S. epidermidis*** **strain 14.1.R1.** (DOCX 19 kb) Additional file 6:
**A. Genome comparison of 17** ***S. epidermidis*** **strains with strain AU23 as reference.** Newly sequenced genomes were included (FS1, AU21, AU24). The other 13 genomes were selected based on their position in the phylogenetic core genome tree (Fig. 2). Regions of genomic variability (1-7) can be detected (see Additional file 7A for their gene content). The BRIG program was used to generate the figure. **B**. Genome comparison of 17 *S. epidermidis* strains with strain FS1 as reference. Newly sequenced genomes were included (AU21, AU23, AU24). The other 13 genomes were selected based on their position in the phylogenetic core genome tree (Fig. 2). Regions of genomic variability can be detected (see Additional file 7B for their gene content). The BRIG program was used to generate the figure. (DOCX 1185 kb) Additional file 7:
**A: Seven genomic islands in strain** ***S. epidermidis*** **AU23.** **B**: Ten genomic islands in strain *S. epidermidis* FS1. **C**: Eight genomic islands in strain *S. epidermidis* 14.1.R1. (XLSX 331 kb) Additional file 8:
**SDS-PAGE analysis of concentrated cell-free supernatant (cCFS) of** ***S. epidermidis*** **14.1.R1.** Different concentrations of the *S. epidermidis* 14.1.R1 cCFS separated on an 18 % polyacrylamide gel and stained with the silver stain method. Lane 1: broad range molecular weight standard. Lanes 2–6: different concentrations of *S. epidermidis* 14.1.R1 cCFS. The gel bands that were analyzed with MALDI-TOF MS are marked A-C. Proteins were identified using Mascot Search of MS/MS data from the peptide digest with databases NCBI-nr, MSDB, and Swiss-Prot (and using the 14.1.R1 genome): A, HMPREF9956_2246 (EsxA) (MW: 11015 Da); B, HMPREF9956_1196 (DNA-binding protein HU (MW: 9626 Da); C, HMPREF9956_0860 and HMPREF9956_0861 (PSMβ1a/1b) (MW: 4639 Da)/HMPREF9956_0859 (PSMβ2) (MW: 4644 Da). (DOCX 368 kb) Additional file 9:
**A. Amino acid sequences of the PSMβ-proteins from** ***S. epidermidis*** **14.1.R1.** The sequence shown in bold red represents the sequence coverage by MS analysis. The sequence coverage was 70 % and 40 % for PSMβ2 and PSMβ1a/1b, respectively. **B**. The *psmβ* operon is composed of four genes in the *S. epidermidis* strain 14.1.R1. The two peptides PSMβ1a (HMPREF9956_0861), and PSMβ1b (HMPREF9956_0860) are identical on protein level; on DNA level there are 3 single nucleotide polymorphisms. (DOCX 26 kb) Additional file 10:
**Amino acid sequence alignment of EsxA from** ***S. epidermidis*** **14.1.R1,** ***S. aureus*** **M0733 and** ***M. tuberculosis*** **H37Rv.** The Trp-Xaa-Gly (WXG) motif, a signature sequence of ESAT-6-like proteins, is marked with three asterisks. The EsxA protein is structurally organized as a helical hairpin, with the conserved WXG motif that localizes in a loop between the two α-helices. (DOCX 82 kb)

## Abbreviations

ADI: arginine deiminase
cCFS: concentrated cell-free supernatant
CDI: contact-dependent growth inhibition
ESAT-6: early secretory antigenic 6 kDa
ESS: ESAT-6 secretion system
MGE: mobile genetic element
SaPI: *S. aureus* pathogenicity island
