# Supplementary material for: Antagonism between Staphylococcus epidermidis and Propionibacterium acnes and its genomic basis
Source: BMC Genomics. 2016 Feb 29;17:152. doi: 10.1186/s12864-016-2489-5 (PMC4770681; doi:10.1186/s12864-016-2489-5)
Supplement: Additional file 1: — P. acnes strains used in this study. (DOCX 35 kb) [file 12864_2016_2489_MOESM1_ESM.docx]

**Additional file 1**. *P. acnes* strains used in this study.

| Phylotype | ST * | strain ** | isolated from | status/disease | origin |
| --- | --- | --- | --- | --- | --- |
| I-1a | 1 | **42.1.R1** | upper back | healthy | Denmark, Aalborg |
| I-1a | 2 | **37.1.R1** | upper back | severe acne | Denmark, Aalborg |
| I-1a | 4 | **19.1.L1** | alar crease | moderate acne | Denmark, Aalborg |
| I-1a | 5 | **14.1.L1** | alar crease | light acne | Denmark, Aalborg |
| I-1a | 6 | **CCUG50480** | n.d. | endocarditis | Sweden, CCUG |
| I-1a | 7 | **40.1.R1** | upper back | moderate acne | Denmark, Aalborg |
| I-1a | 8 | **China 7.1** | n.d. | healthy | China |
| I-1a | 9 | **China 4.1** | n.d. | healthy | China |
| I-1a | 10 | **China 8.1** | n.d. | healthy | China |
| I-1a | 13 | **37.1.L1** | alar crease | severe acne | Denmark, Aalborg |
| I-1a | 14 | **40.1.L1** | alar crease | moderate acne | Denmark, Aalborg |
| I-1a | 15 | **20.2.A1** | face | severe acne | Denmark, Aalborg |
| I-1a | 16 | **21.1.A1** | face | healthy | Denmark, Aalborg |
| I-1a | 17 | **26.2.A1** | face | moderate acne | Denmark, Aalborg |
| I-1a | 18 | **1.4.L1** | alar crease | healthy | Denmark, Aalborg |
| I-1a | 18 | 26.1.R1 | upper back | healthy | Denmark, Aalborg |
| I-1a | 18 | 12.1.A1 | face | n.d. | Denmark, Aalborg |
| I-1a | 18 | 34.2.A1 | face | moderate acne | Denmark, Aalborg |
| I-1a | 18 | 13.1.A1 | face | severe acne | Denmark, Aalborg |
| I-1a | 18 | 20.1.A1 | face | severe acne | Denmark, Aalborg |
| I-1a | 18 | 5.1.A1 | face | n.d. | Denmark, Aalborg |
| I-1a | 18 | 2.4.A1 | face | healthy | Denmark, Aalborg |
| I-1a | 18 | 23.1.A1 | face | healthy | Denmark, Aalborg |
| I-1a | 18 | 32.1.A1 | face | healthy | Denmark, Aalborg |
| I-1a | 19 | **CCUG34938** | n.d. | blood | Sweden, CCUG |
| I-1a | 20 | **12.1.R1** | upper back | moderate acne | Denmark, Aalborg |
| I-1a | 21 | **19.1.R1** | upper back | moderate acne | Denmark, Aalborg |
| I-1a | 22 | **4.4.L1** | alar crease | light acne | Denmark, Aalborg |
| I-1a | 23 | **18.1.R1** | upper back | light acne | Denmark, Aalborg |
| I-1a | 24 | **4.4.R1** | upper back | light acne | Denmark, Aalborg |
| I-1a | 25 | **23.1.L1** | alar crease | healthy | Denmark, Aalborg |
| I-1a | 26 | **China 2.1** | n.d. | healthy | China |
| I-1a | 27 | **1.5.L1** | alar crease | light acne | Denmark, Aalborg |
| I-1a | 27 | 2.1.A2 | face | n.d. | Denmark, Aalborg |
| I-1a | 27 | 3.4.L2 | alar crease | healthy | Denmark, Aalborg |
| I-1a | 27 | 5.1.L1 | alar crease | n.d. | Denmark, Aalborg |
| I-1a | 27 | 24.1.A1 | face | light acne | Denmark, Aalborg |
| I-1a | 27 | 2.1.A2 | face | n.d. | Denmark, Aalborg |
| I-1a | 27 | 8.1.R1 | upper back | n.d. | Denmark, Aalborg |
| I-1a | 27 | 29.1.A1 | face | light acne | Denmark, Aalborg |
| I-1a | 27 | CCUG10171 | n.d. | n.d. | Sweden, CCUG |
| I-1a | 27 | 21.2.R1 | upper back | healthy | Denmark, Aalborg |
| I-1a | 28 | **25.1.R1** | upper back | light acne | Denmark, Aalborg |
| I-1b | 29 | 27.1.R1 | upper back | moderate acne | Denmark, Aalborg |
| I-1b | 30 | 20.2.R1 | upper back | severe acne | Denmark, Aalborg |
| I-1b | 31 | 3.6.A1 | face | healthy | Denmark, Aalborg |
| I-1b | 32 | 16.2.R1 | upper back | light acne | Denmark, Aalborg |
| I-2 | 34 | KPA171202 | n.d. | healthy | Germany, DSMZ |
| I-2 | 35 | 2.3.A1 | face | healthy | Denmark, Aalborg |
| I-2 | 36 | 2.5.A1 | face | healthy | Denmark, Aalborg |
| I-2 | 36 | 6.1.L1 | alar crease | n.d. | Denmark, Aalborg |
| I-2 | 36 | 3.3.R1 | upper back | light acne | Denmark, Aalborg |
| I-2 | 36 | CCUG48138 | n.d. | n.d. | Sweden, CCUG |
| I-2 | 36 | 1.5.A1 | face | light acne | Denmark, Aalborg |
| I-2 | 36 | 16.2.A1 | face | light acne | Denmark, Aalborg |
| I-2 | 36 | 24.1.R1 | upper back | healthy | Denmark, Aalborg |
| I-2 | 36 | 21.1.L1 | alar crease | healthy | Denmark, Aalborg |
| I-2 | 38 | 27.1.A1 | blood | moderate acne | Denmark, Aalborg |
| I-2 | 40 | 27.1.L1 | alar crease | moderate acne | Denmark, Aalborg |
| I-2 | 41 | 36.1.L1 | alar crease | light acne | Denmark, Aalborg |
| I-2 | 42 | CCUG36661 | n.d. | blood | Sweden, CCUG |
| III | 43 | CCUG35900 | n.d. | n.d. | Sweden, CCUG |
| III | 44 | CCUG36986 | n.d. | n.d. | Sweden, CCUG |
| II | 45 | 36.1.R1 | upper back | light acne | Denmark, Aalborg |
| II | 46 | CCUG50655 | n.d. | mandibular gland | Sweden, CCUG |
| II | 47 | 18.2.L1 | alar crease | healthy | Denmark, Aalborg |
| II | 48 | CCUG33951 | n.d. | blood | Sweden, CCUG |
| II | 49 | CHINA 2.3 | blood | healthy | China |
| II | 50 | 7.1.L1 | blood | light acne | Denmark, Aalborg |
| II | 51 | CCUG27534 | n.d. | urinary tract | Sweden, CCUG |
| II | 52 | 5.1.R1 | upper back | healthy | Denmark, Aalborg |
| II | 53 | 18.1.A1 | face | light acne | Denmark, Aalborg |
| II | 53A | CCUG36609 | n.d. | human pustule | Sweden, CCUG |
| II | 54 | 34.1.A1 | face | moderate acne | Denmark, Aalborg |
| II | 55 | CCUG45436 | n.d. | oral cavity | Sweden, CCUG |
| II | 56 | 39.3.R1 | upper back | light acne | Denmark, Aalborg |
| II | 57 | CCUG33206 | n.d. | blood | Sweden, CCUG |

* The MLST scheme of Lomholt and Kilian was used [12].

** The 25 strains that were used as indicator strains are shown in bold.
