# Supplementary material for: Antagonism between Staphylococcus epidermidis and Propionibacterium acnes and its genomic basis
Source: BMC Genomics. 2016 Feb 29;17:152. doi: 10.1186/s12864-016-2489-5 (PMC4770681; doi:10.1186/s12864-016-2489-5)
Supplement: Additional file 2: — S. epidermidis strains used in this study. (DOCX 17 kb) [file 12864_2016_2489_MOESM2_ESM.docx]

**Additional file 2.** *S. epidermidis* strains used in this study.

| strain * | isolated from | status/disease | origin |
| --- | --- | --- | --- |
| AU23 | **nares** | **healthy** | **Denmark, Aalborg** |
| AU24 | nares | healthy | Denmark, Aalborg |
| AU21 | **nares** | **healthy** | **Denmark, Aalborg** |
| AU48 | **nares** | **healthy** | **Denmark, Aalborg** |
| AU35 | nares | healthy | Denmark, Aalborg |
| AS1 | alar crease | light acne | Denmark, Aalborg |
| AU81 | nares | healthy | Denmark, Aalborg |
| AU10 | alar crease | healthy | Denmark, Aalborg |
| AU53 | **nares** | **healthy** | **Denmark, Aalborg** |
| AU73 | nares | healthy | Denmark, Aalborg |
| 14.1.R1 | **upper back** | **light acne** | **Denmark, Aalborg** |
| AU39 | alar crease | healthy | Denmark, Aalborg |
| AU16 | alar crease | healthy | Denmark, Aalborg |
| FS1 | **face** | **light acne** | **Denmark, Aalborg** |
| IS2 | face | light acne | Denmark, Aalborg |
| AU60 | **nares** | **healthy** | **Denmark, Aalborg** |
| AU36 | **nares** | **healthy** | **Denmark, Aalborg** |
| AU44 | nares | healthy | Denmark, Aalborg |
| AU40 | alar crease | healthy | Denmark, Aalborg |
| GS3 | face | moderate acne | Denmark, Aalborg |
| 53.1.A1 | **n.d.** | **n.d.** | **Denmark, Aalborg** |
| AU57 | **nares** | **healthy** | **Denmark, Aalborg** |
| 51.1.A4 | **n.d.** | **n.d.** | **Denmark, Aalborg** |
| 48.1.A1 | **n.d.** | **n.d.** | **Denmark, Aalborg** |

* The 12 strains that were used as indicator strains are shown in bold.
