# Supplementary material for: Antagonism between Staphylococcus epidermidis and Propionibacterium acnes and its genomic basis
Source: BMC Genomics. 2016 Feb 29;17:152. doi: 10.1186/s12864-016-2489-5 (PMC4770681; doi:10.1186/s12864-016-2489-5)
Supplement: Additional file 3: — Inhibitory activity of 77 P. acnes strains against 12 S. epidermidis strains. (DOCX 31 kb) [file 12864_2016_2489_MOESM3_ESM.docx]

**Additional file 3.** Inhibitory activity of 77 *P. acnes* strains against 12 *S. epidermidis* strains.

| *P. acnes*  strain | phylotype | ST | status/disease | antimicrobial activity in %* |
| --- | --- | --- | --- | --- |
| 2.3.A1 | I-2 | 35 | healthy | 75 |
| 27.1.A1 | I-2 | 38 | moderate acne | 75 |
| 2.5.A1 | I-2 | 36 | healthy | 67 |
| 21.1.L1 | I-2 | 36 | healthy | 67 |
| 24.1.R1 | I-2 | 36 | healthy | 67 |
| 6.1.L1 | I-2 | 36 | n.d. | 67 |
| 36.1.L1 | I-2 | 41 | light acne | 67 |
| CCUG36661 | I-2 | 42 | blood | 67 |
| 1.5.A1 | I-2 | 36 | light acne | 58 |
| 16.2.A1 | I-2 | 36 | light acne | 58 |
| CHINA 7.1 | I-1a | 8 | healthy | 50 |
| CHINA 4.1 | I-1a | 9 | healthy | 50 |
| CHINA 8.1 | I-1a | 10 | healthy | 50 |
| 1.4.L1 | I-1a | 18 | healthy | 50 |
| 12.1.R1 | I-1a | 20 | moderate acne | 50 |
| 18.1.R1 | I-1a | 23 | light acne | 50 |
| CHINA 2.1 | I-1a | 26 | healthy | 50 |
| 27.1.R1 | I-1b | 29 | moderate acne | 50 |
| 40.1.R1 | I-1a | 7 | moderate acne | 42 |
| 26.2.A1 | I-1a | 17 | moderate acne | 42 |
| 5.1.A1 | I-1a | 18 | n.d. | 42 |
| CCUG34938 | I-1a | 19 | blood | 42 |
| 19.1.R1 | I-1a | 21 | moderate acne | 42 |
| 3.6.A1 | I-1b | 31 | healthy | 42 |
| 16.2.R1 | I-1b | 32 | light acne | 42 |
| CCUG48138 | I-2 | 36 | n.d. | 42 |
| 37.1R1 | I-1a | 2 | severe acne | 33 |
| 19.1.L1 | I-1a | 4 | moderate acne | 33 |
| 37.1.L1 | I-1a | 13 | severe acne | 33 |
| 40.1.L1 | I-1a | 14 | moderate acne | 33 |
| 20.2.A1 | I-1a | 15 | severe acne | 33 |
| 2.4.A1 | I-1a | 18 | healthy | 33 |
| 23.1.A1 | I-1a | 18 | healthy | 33 |
| 4.4.L1 | I-1a | 22 | light acne | 33 |
| 1.5.L1 | I-1a | 27 | light acne | 33 |
| 2.1.A2 | I-1a | 27 | n.d. | 33 |
| 29.1.A1 | I-1a | 27 | light acne | 33 |
| 3.4.L2 | I-1a | 27 | healthy | 33 |
| 8.1.R1 | I-1a | 27 | n.d. | 33 |
| 20.2.R1 | I-1b | 30 | severe acne | 33 |
| KPA171202 | I-2 | 34 | healthy | 33 |
| 21.1.A1 | I-1a | 16 | healthy | 25 |
| 20.1.A1 | I-1a | 18 | severe acne | 25 |
| 26.1.R1 | I-1a | 18 | healthy | 25 |
| 4.4.R1 | I-1a | 24 | light acne | 25 |
| 23.1.L1 | I-1a | 25 | healthy | 25 |
| 2.1.A2 | I-1a | 27 | n.d. | 25 |
| 24.1.A1 | I-1a | 27 | light acne | 25 |
| 5.1.L1 | I-1a | 27 | n.d. | 25 |
| 25.1.R1 | I-1a | 28 | light acne | 25 |
| 36.1.R1 | II | 45 | light acne | 25 |
| CHINA 2.3 | II | 49 | healthy | 25 |
| CCUG27534 | II | 51 | urinary tract | 25 |
| 18.1.A1 | II | 53 | light acne | 25 |
| 34.1.A1 | II | 54 | moderate acne | 25 |
| CCUG45436 | II | 55 | oral cavity | 25 |
| 39.3.R1 | II | 56 | light acne | 25 |
| CCUG33206 | II | 57 | blood | 25 |
| CCUG36986 | III | 44 | n.d. | 25 |
| 42.1.R1 | I-1a | 1 | healthy | 17 |
| 14.1.L1 | I-1a | 5 | light acne | 17 |
| 12.1.A1 | I-1a | 18 | n.d. | 17 |
| 13.1.A1 | I-1a | 18 | severe acne | 17 |
| 32.1.A1 | I-1a | 18 | healthy | 17 |
| 34.2.A1 | I-1a | 18 | moderate acne | 17 |
| 21.2.R1 | I-1a | 27 | healthy | 17 |
| CCUG10171 | I-1a | 27 | n.d. | 17 |
| 3.3.R1 | I-2 | 36 | light acne | 17 |
| 27.1.L1 | I-2 | 40 | moderate acne | 17 |
| CCUG50655 | II | 46 | mandibular gland | 17 |
| 18.2.L1 | II | 47 | healthy | 17 |
| 7.1.L1 | II | 50 | light acne | 17 |
| CCUG36609 | II | 53A | human pustule | 17 |
| CCUG35900 | III | 43 | n.d. | 17 |
| CCUG50480 | I-1a | 6 | endocarditis | 8 |
| CCUG33951 | II | 48 | blood | 8 |
| 5.1.R1 | II | 52 | healthy | 8 |

* Listed from most potent to least potent strain.
