# Supplementary material for: Antagonism between Staphylococcus epidermidis and Propionibacterium acnes and its genomic basis
Source: BMC Genomics. 2016 Feb 29;17:152. doi: 10.1186/s12864-016-2489-5 (PMC4770681; doi:10.1186/s12864-016-2489-5)
Supplement: Additional file 4: — Sensitivity of individual P. acnes strains against the antimicrobial activity of 20 S. epidermidis strains. (DOCX 16 kb) [file 12864_2016_2489_MOESM4_ESM.docx]

**Additional file 4.** Sensitivity of individual *P. acnes* strains against the antimicrobial activity of 20 *S. epidermidis* strains

| **Strain name and ST (CC18 isolates)** | **Strain origin** | ***S. epidermidis* antimicrobial activity *** | **Strain name and ST (other I-1a isolates)** | **Strain origin** | ***S. epidermidis* antimicrobial activity *** |
| --- | --- | --- | --- | --- | --- |
| **21.1.A1 (ST16)** | Healthy | 8 | **42.1.R1 (ST1)** | Healthy | 9 |
| **40.1.L1 (ST14)** | Moderate | 7 | **37.1R1 (ST2)** | Severe | 8 |
| **CCUG50480 (ST6)** | Healthy | 6 | **19.1.L1 (ST4)** | Moderate | 7 |
| **12.1.R1 (ST20)** | Moderate | 6 | **25.1.R1 (ST28)** | Light | 7 |
| **20.2.A1 (ST15)** | Severe | 6 | **4.4.L1 (ST22)** | Light | 7 |
| **14.1.L1 (ST5)** | Light | 5 | **4.4.R1 (ST24)** | Light | 6 |
| **37.1.L1 (ST13)** | Severe | 5 | **18.1.R1 (ST23)** | Light | 5 |
| **CHINA 8.1 (ST10)** | Healthy | 5 | **CHINA 2.1 (ST26)** | Healthy | 5 |
| **1.4.L1 (ST18)** | Healthy | 4 | **19.1.R1 (ST21)** | Moderate | 5 |
| **CCUG34938 (ST19)** | Healthy | 4 | **23.1.L1 (ST25)** | n.d. | 4 |
| **CHINA 4.1 (ST9)** | Healthy | 3 | **1.5.L1 (ST27)** | Light | 2 |
| **40.1.R1 (ST7)** | Light | 3 |  |  |  |
| **CHINA 7.1 (ST8)** | Healthy | 3 |  |  |  |
| **26.2.A1 (ST17)** | Moderate | 2 |  |  |  |

* number of *S. epidermidis* strains (out of 20 tested) that inhibited a given *P. acnes* strain
