# Supplementary material for: Antagonism between Staphylococcus epidermidis and Propionibacterium acnes and its genomic basis
Source: BMC Genomics. 2016 Feb 29;17:152. doi: 10.1186/s12864-016-2489-5 (PMC4770681; doi:10.1186/s12864-016-2489-5)
Supplement: Additional file 5: — Antimicrobial activity spectrum of S. epidermidis strain 14.1.R1. (DOCX 19 kb) [file 12864_2016_2489_MOESM5_ESM.docx]

**Additional file 5.** Antimicrobial activity spectrum of *S. epidermidis* strain 14.1.R1.

| Indicator species | Susceptible to  *S. epidermidis* 14.1.R1 |
| --- | --- |
| *P. acnes* strains and ST * |  |
| 27.1.R1 (ST29) | + |
| 20.2.R1 (ST30) | + |
| 3.6.A1 (ST31) | + |
| 16.2.R1 (ST32) | + |
| KPA171202 (ST34) | + |
| 2.3.A1 (ST35) | + |
| 21.2.A1 (ST37) | + |
| 27.1.A1 (ST38) | + |
| 27.1.L1 (ST40) | + |
| 36.1.L1 (ST41) | + |
| CCUG36661 (ST42) | + |
| CCUG35900 (ST43) | + |
| CCUG36986 (ST44) | + |
| 36.1.R1 (ST45) | + |
| CCUG50655 (ST46) | + |
| 18.2.L1 (ST47) | + |
| CCUG33951 (ST48) | + |
| CHINA 2.3 (ST49) | + |
| 7.1.L1 (ST50) | + |
| CCUG27534 (ST51) | + |
| 5.1.R1 (ST52) | + |
| 18.1.A1 (ST53) | + |
| CCUG36609 (ST53) | + |
| 34.1.A1 (ST54) | + |
| CCUG45436 (ST55) | + |
| 39.3.R1 (ST56) | + |
| CCUG33206 (ST57) | + |
| *Propionibacterium granulosum* | + |
| *Staphylococcus aureus* | - |
| *Enterococcus faecalis* | - |
| *Candida albicans* | - |
| *Streptococcus pneumoniae* | - |
| *Klebsielle pneumoniae* | - |
| *Streptococcus pyogenes* | - |
| *Pseudomonas aeruginosa* | - |
| *Staphylococcus epidermidis* | - |
| *Proteus mirabilis* | - |
| *Escherichia coli* | - |
| *Salmonella ariconae* | - |

* see Additional file 1 for strain origin
