# Supplementary material for: Antagonism between Staphylococcus epidermidis and Propionibacterium acnes and its genomic basis
Source: BMC Genomics. 2016 Feb 29;17:152. doi: 10.1186/s12864-016-2489-5 (PMC4770681; doi:10.1186/s12864-016-2489-5)
Supplement: Additional file 6: — A. Genome comparison of 17 S. epidermidis strains with strain AU23 as reference. Newly sequenced genomes were included (FS1, AU21, AU24). The other 13 genomes were selected based on their position in the phylogenetic core genome tree (Fig. 2). Regions of genomic variability (1-7) can be detected (see Additional file 7A for their gene content). The BRIG program was used to generate the figure. B. Genome comparison of 17 S. epidermidis strains with strain FS1 as reference. Newly sequenced genomes were included (AU21, AU23, AU24). The other 13 genomes were selected based on their position in the phylogenetic core genome tree (Fig. 2). Regions of genomic variability can be detected (see Additional file 7B for their gene content). The BRIG program was used to generate the figure. (DOCX 1185 kb) [file 12864_2016_2489_MOESM6_ESM.docx]

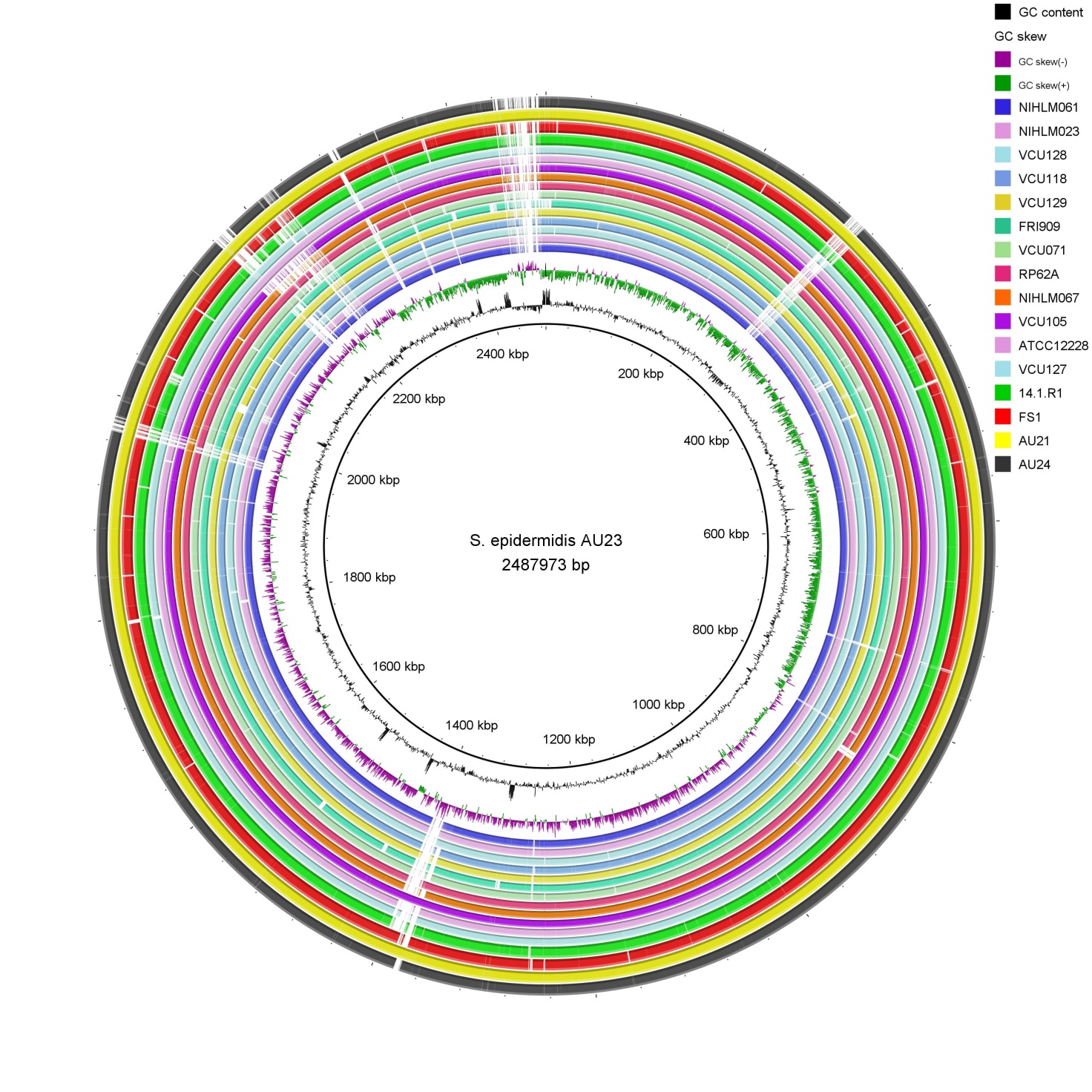


1

7

6

5

4

3

2

**Additional file 6A.** **Genome comparison of 17 *S. epidermidis* strains with strain AU23 as reference**

Newly sequenced genomes were included (FS1, AU21, AU24). The other 13 genomes were selected based on their position in the phylogenetic core genome tree (Figure 2).Regions of genomic variability (1-7) can be detected (see Additional file 7A for their gene content). The BRIG program was used to generate the figure.


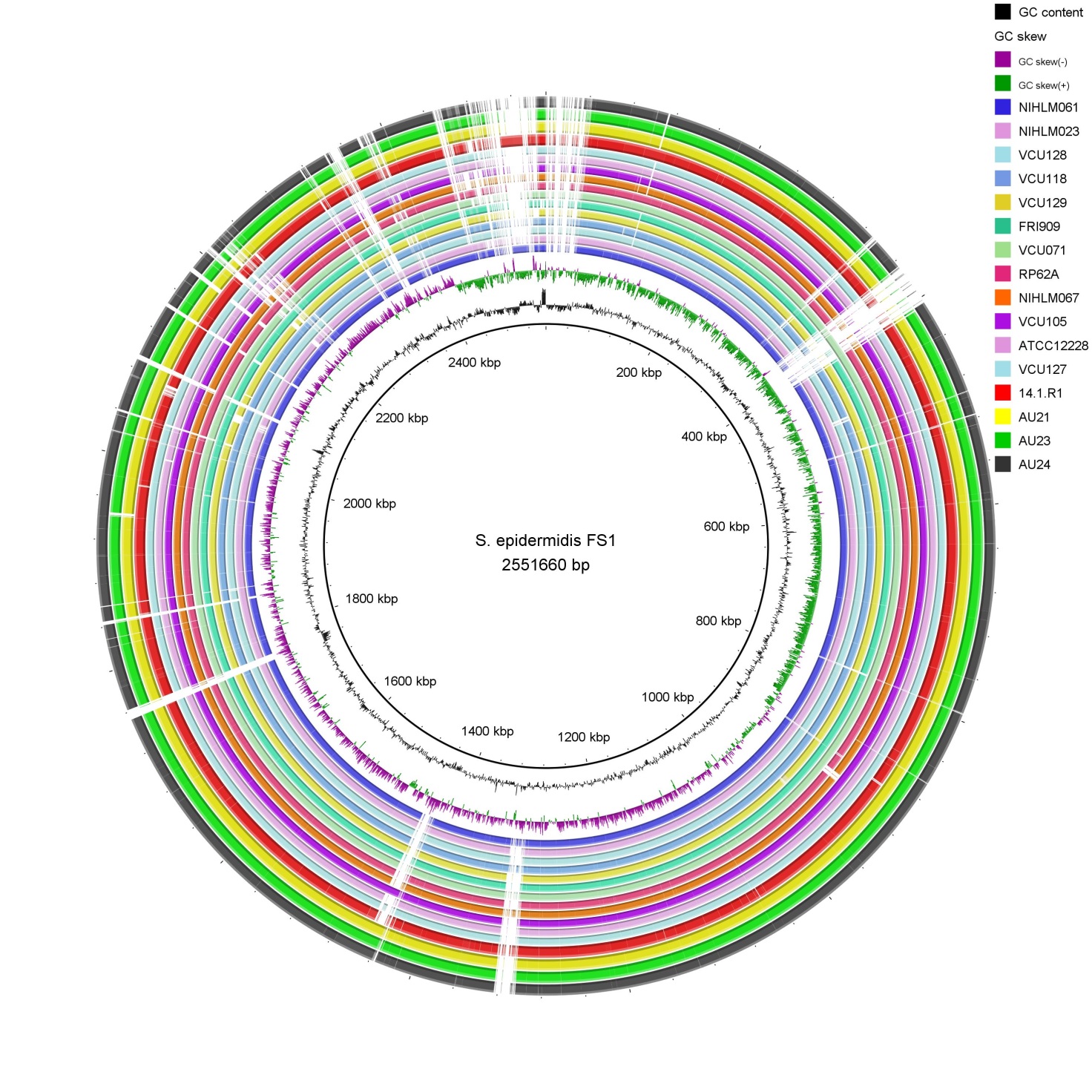


8

1

10

9

7

4

3

2

6

5

**Additional file 6B.** **Genome comparison of 17 *S. epidermidis* strains with strain FS1 as reference**

Newly sequenced genomes were included (AU21, AU23, AU24). The other 13 genomes were selected based on their position in the phylogenetic core genome tree (Figure 2). Regions of genomic variability can be detected (see Additional file 7B for their gene content). The BRIG program was used to generate the figure.
