# Supplementary material for: Antagonism between Staphylococcus epidermidis and Propionibacterium acnes and its genomic basis
Source: BMC Genomics. 2016 Feb 29;17:152. doi: 10.1186/s12864-016-2489-5 (PMC4770681; doi:10.1186/s12864-016-2489-5)
Supplement: Additional file 8: — SDS-PAGE analysis of concentrated cell-free supernatant (cCFS) of S. epidermidis 14.1.R1. Different concentrations of the S. epidermidis 14.1.R1 cCFS separated on an 18 % polyacrylamide gel and stained with the silver stain method. Lane 1: broad range molecular weight standard. Lanes 2–6: different concentrations of S. epidermidis 14.1.R1 cCFS. The gel bands that were analyzed with MALDI-TOF MS are marked A-C. Proteins were identified using Mascot Search of MS/MS data from the peptide digest with databases NCBI-nr, MSDB, and Swiss-Prot (and using the 14.1.R1 genome): A, HMPREF9956_2246 (EsxA) (MW: 11015 Da); B, HMPREF9956_1196 (DNA-binding protein HU (MW: 9626 Da); C, HMPREF9956_0860 and HMPREF9956_0861 (PSMβ1a/1b) (MW: 4639 Da)/HMPREF9956_0859 (PSMβ2) (MW: 4644 Da). (DOCX 368 kb) [file 12864_2016_2489_MOESM8_ESM.docx]

**Additional file 8: SDS-PAGE analysis of concentrated cell-free supernatant (cCFS) of *S. epidermidis* 14.1.R1**

Different concentrations of the *S. epidermidis* 14.1.R1 cCFS separated on an 18% polyacrylamide gel and stained with the silver stain method. Lane 1: broad range molecular weight standard. Lanes 2–6: different concentrations of *S. epidermidis* 14.1.R1 cCFS. The gel bands that were analyzed with MALDI-TOF MS are marked A-C. Proteins were identified using Mascot Search of MS/MS data from the peptide digest with databases NCBI-nr, MSDB, and Swiss-Prot (and using the 14.1.R1 genome): A, HMPREF9956_2246 (EsxA) (MW: 11015 Da); B, HMPREF9956_1196 (DNA-binding protein HU (MW: 9626 Da); C, HMPREF9956_0860 and HMPREF9956_0861 (PSMβ1a/1b) (MW: 4639 Da)/ HMPREF9956_0859 (PSMβ2) (MW: 4644 Da).
