# Supplementary material for: Antagonism between Staphylococcus epidermidis and Propionibacterium acnes and its genomic basis
Source: BMC Genomics. 2016 Feb 29;17:152. doi: 10.1186/s12864-016-2489-5 (PMC4770681; doi:10.1186/s12864-016-2489-5)
Supplement: Additional file 9: — A. Amino acid sequences of the PSMβ-proteins from S. epidermidis 14.1.R1. The sequence shown in bold red represents the sequence coverage by MS analysis. The sequence coverage was 70 % and 40 % for PSMβ2 and PSMβ1a/1b, respectively. B. The psmβ operon is composed of four genes in the S. epidermidis strain 14.1.R1. The two peptides PSMβ1a (HMPREF9956_0861), and PSMβ1b (HMPREF9956_0860) are identical on protein level; on DNA level there are 3 single nucleotide polymorphisms. (DOCX 26 kb) [file 12864_2016_2489_MOESM9_ESM.docx]

MEQLFDAIR**SVVDAGINQDWSQLASGIAGIVENGISVISK**LLGQ (psmβ2)

MSKLAEAIANTVKAAQDQDW**TKLGTSIVDIVESGVSVLGK**IFGF (psmβ1a/1b)

MKLFNAFKDILEAAITNDGTQLGASIVNIIESSVDMVNRFLGN (psmβ3)

**Additional file 9A. Amino acid sequences of the PSMβ-proteins from *S. epidermidis* 14.1.R1**

The sequence shown in bold red represents the sequence coverage by MS analysis. The sequence coverage was 70% and 40% for PSMβ2 and PSMβ1a/1b, respectively.

Nucleotidase

(YjjG family)

Nucleoside

triphosphatase

Phospho-esterase

tRNA

β3 β2 β1a β1b

**Additional file 9B. The *psmβ* operon is composed of four genes in the *S. epidermidis* strain 14.1.R1.**

The two peptides PSMβ1a (HMPREF9956_0861), and PSMβ1b (HMPREF9956_0860) are identical on protein level; on DNA level there are 3 single nucleotide polymorphisms.
