# Supplementary material for: Antagonism between Staphylococcus epidermidis and Propionibacterium acnes and its genomic basis
Source: BMC Genomics. 2016 Feb 29;17:152. doi: 10.1186/s12864-016-2489-5 (PMC4770681; doi:10.1186/s12864-016-2489-5)
Supplement: Additional file 10: — Amino acid sequence alignment of EsxA from S. epidermidis 14.1.R1, S. aureus M0733 and M. tuberculosis H37Rv. The Trp-Xaa-Gly (WXG) motif, a signature sequence of ESAT-6-like proteins, is marked with three asterisks. The EsxA protein is structurally organized as a helical hairpin, with the conserved WXG motif that localizes in a loop between the two α-helices. (DOCX 82 kb) [file 12864_2016_2489_MOESM10_ESM.docx]

**Additional file 10. Amino acid sequence alignment of EsxA from *S. epidermidis* 14.1.R1, *S. aureus* M0733 and *M. tuberculosis* H37Rv.** The Trp-Xaa-Gly (WXG) motif, a signature sequence of ESAT-6-like proteins, is marked with three asterisks. The EsxA protein is structurally organized as a helical hairpin, with the conserved WXG motif that localizes in a loop between the two α-helices.
